# Iron-binding characterization and polysaccharide production by *Klebsiella oxytoca* strain isolated from mine acid drainage

**DOI:** 10.1111/j.1365-2672.2009.04302.x

**Published:** 2009-10

**Authors:** F Baldi, D Marchetto, D Battistel, S Daniele, C Faleri, C De Castro, R Lanzetta

**Affiliations:** 1 Department of Environmental Science, Cà Foscari University of VeneziaVenezia, Italy; 2 Department of Physical Chemistry, Cà Foscari University of VeneziaVenezia, Italy; 3 Department of Environmental Science ‘G. Sarfatti’, University of SienaSiena, Italy; 4 Department of Organic Chemistry and Biochemistry, University of Naples Federico IINaples, Italy

**Keywords:** capsule, citrate fermentation, cyclic voltammetry, exopolysaccharide, iron binding

## Abstract

**Aims:**

To investigate *Klebsiella oxytoca* strain BAS-10 growth on ferric citrate under anaerobic conditions for exopolysaccharide (EPS) production and localization on cell followed by the purification and the EPS determination of the iron-binding stability constant to EPS or biotechnological applications.

**Methods and Results:**

*Klebsiella oxytoca* ferments ferric citrate under anaerobic conditions and produces a ferric hydrogel, whereas ferrous ions were formed in solution. During growth, cells precipitate and a hydrogel formation was observed: the organic material was constituted of an EPS bound to Fe(III) ions, this was found by chemical analyses of the iron species and transmission electron microscopy of the cell cultures. Iron binding to EPS was studied by cyclic voltammetric measurements, either directly on the hydrogel or in an aqueous solutions containing Fe(III)-citrate and purified Fe(III)-EPS. From the voltammetric data, the stability constant for the Fe(III)-EPS complex can be assumed to have values of approx. 10^12^–10^13^. It was estimated that this is higher than for the Fe(III)-citrate complex.

**Conclusions:**

The production of Fe(III)-EPS under anaerobic conditions is a strategy for the strain to survive in mine drainages and other acidic conditions. This physiological feature can be used to produce large amounts of valuable Fe(III)-EPS, starting from a low cost substrate such as Fe(III)-citrate.

**Significant and Impact of the Study:**

The data herein demonstrates that an interesting metal-binding molecule can be produced as a novel catalyst for a variety of potential applications and the EPS itself is a valuable source for rhamnose purification.

## Introduction

Iron is one of the major limiting nutrients and most of the time this element is not available in the ecosystem because it forms oxy-hydroxides (Schröder *et al.* 2003). There are instead habitats where the abundance of iron is visibly high because of a rusty colour owing to the large amount of ferric hydroxides. This is the case of acid drainage waters from pyrite mines, in which, iron represents one of the major elements in solution. In this case, iron is not limiting, but it can represent an environmental hazard for life, especially when it is associated with Cd, Cu, Hg, Pb, Zn and other metals that may be leached out from ore deposits. In such ecosystems, the presence of metals increases the total toxicity of mine drainages, causing a significant reduction of microbial biodiversity (Johnson and Hallberg 2003). This feature, in combination with natural oligotrophism of acid mine drainages, plays an important role in selecting chemolithotrophs (Gonzales-Toril *et al.* 2003; Diaby *et al.* 2007). Conversely, *Enterobacteraceae* are not expected to survive in such acidic ecosystems with high heavy metal concentrations and carbon-depleted habitats. Nevertheless, several years ago, a *Klebsiella oxytoca* strain BAS-10 was isolated under an iron mat formed by waters leached from pyrite mine drainages of Colline Metallifere, Tuscany, Italy (Baldi *et al.* 2001).

*Klebsiella* genus is, in general, responsible for nosocomial infection and is the second most frequently encountered species, and the BAS-10 strain was the first isolate from a mining environment (Baldi *et al.* 2001). Despite this hostile habitat for *Klebsiella*, BAS-10 strain ferments high concentrations of ferric citrate as a sole energy and carbon source under anaerobic conditions (Baldi *et al.* 2001). Citrate is ubiquitous in nature and many bacteria could synthesize this organic compound (Holmes *et al.* 2005) and others have a specific transport system for it (Korithoski *et al.* 2005). Citrate can also be considered the simplest siderophore, capable of transporting iron into cells. *Klebsiella pneumoniae*, with the well-known *Cit*-operon, possesses two distinct citrate transporters: a proton-dependent CitH transporter under aerobic conditions (Bott 1997) and a CitS, a sodium ion-dependent transporter, under anaerobic conditions (Lolkema *et al.* 1994). Citrate is used anaerobically by a narrow spectrum of micro-organisms because the tricarboxylic acid cycle (TCA) is repressed at the step of oxoglutarate-dehydrogenase; so citrate-fermenting bacteria must have a *Cit*-operon (Dimroth 1997) synthesizing a citrate lyase. *Cit*-operon sequences are well known and exist in many other related species (Bott and Dimroth 1994). During citrate fermentation, this compound is transported into the cell through a sodium-dependent carrier coupled to the transport of two sodium ions (Lolkema *et al.* 1994). *Klebsiella oxytoca* strain BAS-10 grows also in 50 mmol l^−1^ ferric citrate and produces a thick iron hydrogel, suggesting a coupled transport of citrate with the ferric ion. Recently, the structural analysis and its heptameric repeating unit of this Fe(III)-binding exopolysaccharide (EPS) has been characterized by spectroscopic methods (Leone *et al.* 2007). One of the *Klebsiella* genus peculiarities is the production of diverse types of capsular polysaccharides, which have been studied mainly in terms of the antigenic specificity of O- and K-antigens (Roberts 1996) and about 80 serotypes have been classified (Mori *et al.* 1989). Each polysaccharide differs in the kind of sequence or linkage to glycosides (Kobayashi *et al.* 2002).

In recent years, there has been interest in polysaccharides because of their biodegradability in the ecosystem. In addition, there is a strong interest in the development of nano-composites resins (Wang *et al.* 1998) and more conventional processes such as purifying metal-binding organic molecules for use as catalysts for selective hydroxylation (Zhang *et al.* 2000).

The aim of this work was to investigate further *Kl. oxytoca* strain BAS-10 growth on ferric citrate under anaerobic conditions for EPS production and localization on cell, followed by purification, determination and calculation of iron-binding stability constant of EPS for various applications. From a biotechnological point of view, it might be important also to understand the mechanism of ferric hydrogel formation and its role in the physiology of the strain living in high iron concentrations.

## Materials and methods

### Media preparation

*Klebsiella oxytoca* BAS-10 was cultured aerobically on complex medium Nutrient Broth (Difco). The strain, depending on experiments, was also cultured anaerobically on three different synthetic media: the first medium contains per litre: 2·5 g NaHCO_3_, 1·5 g NH_4_Cl, 0·6 g NaH_2_PO_4_, 0·1 g KCl with 50 mmol l^−1^ ferric citrate (hereafter referred as FeC medium); the second medium contains the same basic salts with 50 mmol l^−1^ Na-citrate (hereafter referred as NaC medium). Both media were buffered at pH 7·4 with NaOH 1 N. The third medium was prepared by substituting Na-salts with KHCO_3_, KHCO_3_ and KH_2_PO_4_, with 50 mmol l^−1^ ferric citrate and finally was buffered at pH 7·4 with KOH 1 N (hereafter referred to FeKC medium). Citrates were consumed as energy and carbon sources. Anaerobic conditions were maintained in sealed vials with 100 ml medium previously boiled for 5 min to degas O_2_. The medium was cooled to room temperature under N_2_ flux and the vial was sealed.

### Culture conditions

*Klebsiella oxytoca* BAS-10 was inoculated (1 : 100; v/v) on complex medium Nutrient Broth (Difco) starting from frozen culture stock maintained at −80°C in glycerol (20%) and cultured aerobically at 30°C. An aliquot of 1 ml of this aerobic culture was centrifuged and washed with physiological solution (9‰ NaCl) and suspended in the same solution. The inoculum 1 : 100 (v/v; 3·1 ± 0·2 mg protein per ml) was injected with a syringe in the sealed vials containing different citrate media and incubated anaerobically in the dark at 30°C. Samples in double replicates were taken at different times for chemical and biological determinations. Cell biomass was determined as total proteins by the Coomassie Blue method (Bradford 1976) against serum albumin standards. The coefficient of variation of five replicates was 1·2%. The cells were counted under an epifluorescence microscope after staining specimens with 4,6-diamidino-2-phenylindole (final concentration 1 *μ*g ml^−1^) after 15 min in the dark. Other *Kl. oxytoca* strains were cultivated in anaerobic conditions and compared with the BAS-10 strain grown in NaC and FeC media; these strains, 17A24 and 18D29, were kindly donated by the Department for Infectious Diseases and strain KI046 by the Microbiology Section of Molecular Biology Department (University of Siena).

### Carbon dioxide production

The CO_2_ production during Fe-citrate fermentation was determined by an automated OxiTop® control system (Wissenschaftlich-Technische Werkstaetten, Weilheim, Germany), in sealed reaction vessels with pressure sensors, kept at constant temperature (28°C). Fe-citrate (100 ml), placed in a 1-l vessel, were inoculated with *Kl. oxytoca* BAS-10 (2·86 ± 0·46 mg protein) and the pressure increase (hPa) in the headspace of the reaction vessel was recorded every 20 min by remote sensing. Pressure was recorded as hPa using the equation of the OxiTop® protocol. In the control experiment, the growth medium did not contain ferric citrate but only mineral salts.

### Determination of total iron and Fe(II)

A 3-ml aliquot of BAS-10 culture was collected at different times from inoculated and uninoculated (control) vials. Iron species were determined in all samples and analysed in subsamples after shaking the cultures to obtain a homogeneous emulsion and without shaking to determine the iron species, especially when the hydrogel was precipitated. Total iron concentration was determined as total Fe(II), after reducing all ferric ions with hydroxylamine, followed by colorimetric determination using the *o*-phenanthroline method (ASTM 1986). The red-coloured solution produced by the reaction of *o*-phenanthroline with Fe(II) was measured with a UV–visible spectrophotometer (Shimadzu UV-160; Shimadzu, Kyoto, Japan) at 510 nm. The Fe(II) species was determined after shaking the sample and filtering through a 0·2-*μ*m sterile filter; the effective reduced Fe(II) was determined without addition of hydroxylamine to the sample. The total amount of Fe(III) was determined by difference of the total iron and Fe(II) content without hydroxylamine additions.

An experiment was also performed to test the Fe(II) formation of BAS-10 with Fe-citrate as the sole carbon source in the presence of sodium or potassium salts, namely in the FeC and FeKC media.

### EPS determination and purification

EPS was quantified in the culture medium as glucuronic acid equivalents after reaction with sodium tetraborate 0·0125 mol l^−1^ in H_2_SO_4_ (100°C, 6 min), the addition of *m*-hydroxydiphenyl and spectrophotometric measurement at 520 nm (Blumenkrantz and Asboe-Hansen 1973). The concentration of total carbohydrate was quantified as glucose equivalents after reaction with 96% sulfuric acid and 5% phenol and was followed spectrophotometrically at 485 nm (Dubois *et al.* 1956). Iron removal from polysaccharide was performed by treating the rusty gel with EDTA (100 mmol l^−1^) until it was colourless. The measurements of the polysaccharide were performed after iron had been removed by performing several extractions with an EDTA solution (100 mmol l^−1^) until the rusty gel became colourless. Finally, iron residues were completely removed from EPS by gel permeation chromatography and the EPS extract was freeze-dried (Leone *et al.* 2007).

### Determination of total iron in purified EPS

The culture was incubated in 100 ml FeC medium, after formation of the hydrogel, Fe(III)-EPS was recovered and purified by centrifugation to eliminate cells. The gel was precipitated for three times with cold ethanol (70%) and was maintained for each step overnight at −20°C. The Fe(III)-EPS was dried under vacuum. Three different aliquots of about 30 mg of solid polymer were acidified with 2 ml of concentrated HNO_3_ and made up to 100 ml with high-resistance deionized water. Total iron was analysed in triplicate by flame AAS (Varian Spectra AA, model 250; Varian, Wilmington, DE).

### Transmission electron microscope observations

To observe the iron in EPS and cell envelope conditions, *Kl. oxytoca* BAS-10 cells were grown in FeC medium after 6 days of incubation at 30°C. During cell growth, several specimens were prepared for transmission electron microscopy (TEM), before and after hydrogel precipitation, at 6 and 10 days, respectively. Cells were harvested by centrifugation at 8100 ***g***. The bacterial pellet was fixed for 1 h at 4°C with 2·5% glutaraldehyde and 0·1 mol l^−1^ lysine in 0·066 mol l^−1^ cacodylate buffer, at pH 7·2, for 30 min at room temperature. The cells were washed five times in the same buffer and postfixed for 1 h at room temperature in 1% osmium tetroxide, rinsed with distilled water, and embedded in Spurr resin. Ultrathin sections were prepared using a LKB II Nova Ultramicrotome (LKB, Victoria, Australia) with a diamond knife. Sections were stained with 3·0% uranyl acetate solution for 15 min, washed once with distilled water and incubated in lead citrate for 10 min. TEM observations were performed with a JEOL JEM 100b (Jeol, Tokyo, Japan) operating under standard conditions.

### Voltammetric measurements and simulations

Voltammetric experiments were performed to estimate the relative stability constant of the EPS-Fe(III) complex with respect to the Fe(III)-citrate complex. The voltammetric measurements were carried out in a two-electrode electrochemical cell, which was located in a Faraday cage made of aluminium sheets to avoid external noise. The working electrode was a mercury-coated Pt microdisk of 25-*μ*m diameter. The Pt microdisk was prepared by sealing a Pt wire of 25-*μ*m diameter, directly in glass (Baldo *et al.* 1995). The mercury film was produced by cathodic deposition of mercury onto the platinum microdisk under potentiostatic conditions in a plating solution consisting of 5 mmol l^−1^ Hg_2_(NO_3_)_2_ in 1 mmol l^−1^ KNO_3_ acidified with nitric acid to pH <1 (Baldo *et al.* 1995). The reference/counter electrode was either an Ag/AgCl electrode saturated with KCl or a Pt wire, which was used as pseudo reference electrode (PtPRE). The Pt working electrode was polished mechanically with graded alumina powder (1, 0·3 *μ*m down to 0·05 *μ*m) on a polishing microcloth. Cyclic voltammetric and potentiostatic experiments were performed by using a potentiostat/galvanostat PAR 283 A (EG&G, Princeton, NJ) and was controlled via the 270 PAR (EG&G) software on a personal computer. Unless otherwise stated, steady-state voltammograms with the microelectrode were recorded at 10 mV s^−1^ and at room temperature (21 ± 1°C). The electrode process involving both the Fe-EPS and Fe-citrate systems was simulated with the commercial package DigiSim® (ver. 3.3, Bioanalytical Systems, West Lafayette, IN). The microelectrode behaviour was modelled by assuming hemispherical geometry with semi-infinite diffusion (Bard and Faulkner 2001). On the basis of the experimental results (vide infra), the heterogeneous electron transfer (Bard and Faulkner 2001) for the reduction of Fe(III)-EPS and Fe(III)-citrate complexes were assumed to be not reversible, and values of the rate constants in the range 10^−4^–10^−5^ cm s^−1^ were used in the simulations.

## Results

### Cell growth and polysaccharide production

A comparison of four different strains of *Kl. oxytoca* with different environmental origins was performed under anaerobic conditions to demonstrate that ferric citrate fermentation is a specific characteristic of BAS-10 strain (Table 1). The biomass in terms of total proteins for each strain was measured in triplicate in NaC and FeC media after 2 days of incubation. Three strains could not grow on Fe(III)-citrate because iron seems inhibited citrate fermentation in all strains except BAS-10.

**Table 1:** Growth comparisons of different strains of *Klebsiella oxytoca* of different origin inoculated in NaC and FeC media

		Growth	
Strains	Origin	NaC	FeC
BAS-10	This study	++	++
KI046	Molecular Biology Department	w	−
17A24	Infection Diseases Department	++	−
18D29	Infection Diseases Department	++	w

−, nongrowth; w, weak growth; ++, abundant growth.

**Figure 1:**
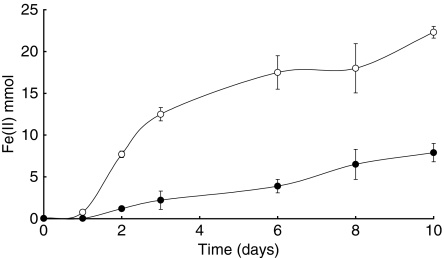
Fe(II) production by *Klebsiella oxytoca* BAS-10 from Fe(III)-citrate fermentation in FeC ( • ) and in FeKC ( ○ ) media with and without sodium additions, respectively. Bar errors are from double replicates Fe(II) determinations.

BAS-10 strain grown on FeC medium was characterized by the formation of a ferric hydrogel, whereas it was not formed in a NaC medium. BAS-10 during citrate fermentation under anaerobic conditions at 30°C shown 2·31 generations per day in NaC, whereas in FeC medium, this was 1·84 day^−1^. After 2 days of growth, a decrease in cell numbers and proteins occurred in FeC medium, but a significant regression line between protein (PRT) and cell number was maintained during the 10 days of the experiments: 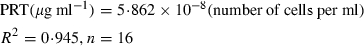


The presence of Na^+^ or Fe(III) complexes with citrate changed the physiology of the BAS-10 strain. The role of iron in Fe(III)-citrate fermentation was tested by determining Fe(II) production during BAS-10 growth in sealed vials in FeC and FeKC media, respectively, with sodium and without sodium additions in shaken vials (Fig. 1). BAS-10 showed a Fe(II) production that was higher than in media without sodium additions. The reduction of Fe(III) to Fe(II) from ferric citrate was up to 44·7% in FeKC medium, whereas it was lower (by 10%) in FeC medium at the end of the experiment. So citrate fermentation was driven by Fe(III) even in the absence of Na^+^, although the latter is preferred in citrate fermentation.

**Figure 2:**
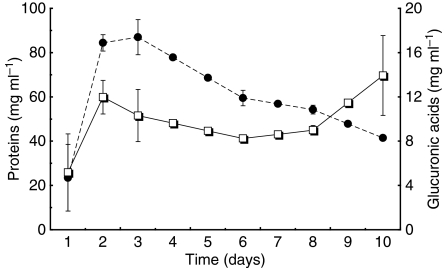
Correlation found between proteins (*μ*g ml^−1^) ( • ) and glucuronic equivalents (*μ*g ml^−1^) ( ○ ) as an indicator of polysaccharide production, determined in cell pellets during 10 days of incubation in FeC medium under anaerobic conditions. Bar errors are from double replicates analyses.

Proteins formation *vs* EPS production by BAS-10 cells followed a similar trend, for 6 days of the experiments, and then EPS started increasing from 8 up to 14 *μ*g ml^−1^ (Fig. 2). This late EPS production was concomitant with iron hydrogel precipitation. This effect was more evident if the total polysaccharide/protein (CHO/PRT) ratio was calculated and compared with the total iron in solution, in a nonshaken culture, when the hydrogel was forming, although, the total Fe remains constant at 49·86 ± 0·817 mmol l^−1^ in shaken and hence homogenized cultures. The CHO/PRT ratio remained constant for 6 days at 2·19 ± 0·327 and than it started increasing to 4 (8 days) and up to 5·73 after 10 days of incubation. While in not-shaken culture total Fe dropped down to 8 mmol l^−1^ with the hydrogel.

**Figure 3:**
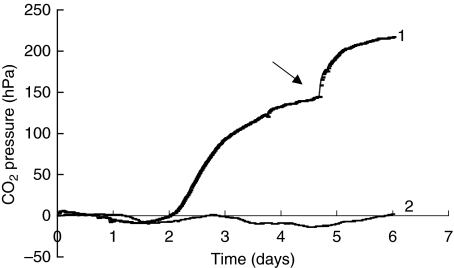
Determination of CO_2_ pressure (hPa) in a sealed 1-l Oxytop bioreactor (plot 1) during citrate fermentation by 100-ml culture of *Klebsiella oxytoca* BAS-10 and its uninoculated control (plot 2). After ferric hydrogel was precipitated, the culture was shaken (arrow) by magnetic stirring to remove gas entrapped by ferric hydrogel.

The growth of BAS-10 was followed also by determination of the CO_2_ formed during citrate fermentation and hydrogel formation. The gas emission in a 1-l bioreactor reached a pressure of 144 hPa in nonshaken cultures after incubation at 30°C in FeC medium, with a production rate of 2·82 hPa CO_2_ per h per *μ*g protein (Fig. 3) *vs* a noninoculated sample. After 2 days of lag-phase, the CO_2_ levelled off after 4 days; at this point, the BAS-10 culture (100 ml) was continuously shaken by magnetic stirring and released a further 33·6% of the CO_2_ that was adsorbed on the hydrogel, the final pressure reached up to 217 hPa (Fig. 3 see arrow).

### TEM observations of cells in ferric hydrogel

**Figure 4:**
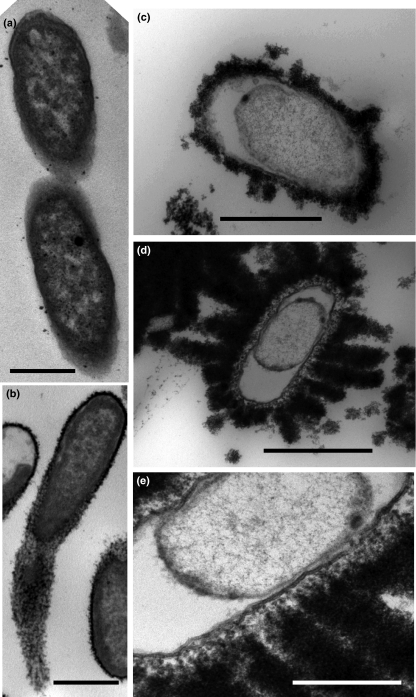
Transmission electron microscopy observations of *Klebsiella oxytoca* BAS-10 (a) growing anaerobically in NaC medium; bar = 1 *μ*m. (b) High electronic dense iron-binding capsular EPS of a cell growing anaerobically in FeC medium. Bar 1 *μ*m was for (a) and (b) photos. (c) A short rod of *Kl. oxytoca* BAS-10 with detached inner membrane from wall and with very electron dense extrusion such as a bud of Fe(III)-EPS; bar = 0·5 *μ*m. (d) A similar cell but with an overwhelmed production of Fe(III)-EPS like plumes; bar = 1 *μ*m; (e) a detail of EPS emission in some spotted zones of envelope and formation of nano-vesicles out of outer membrane; bar = 0·5 *μ*m.

TEM was performed to determine where iron was localized in the BAS-10 cells. Many observations were made of BAS-10 cells during growth in NaC and FeC medium during capsular EPS formation, followed by hydrogel precipitation. After a few days of incubation, *Kl. oxytoca* BAS-10 produces a capsular EPS even in NaC medium (Fig. 4a), but in FeC medium, the capsular EPS was wider and more electron dense because of iron binding (Fig. 4b).

After 10 days, when the ferric hydrogel was precipitated, cells showed a thicker electron dense capsule (Fig. 4c–e), many short-rods were found and differed from the typical long rod cells of *Kl. oxytoca*. A hyperproduction of iron-binding polysaccharide was secreted sometime such as a bud from the cell envelope (Fig. 4c). The new morphology of the cell was characterized by a detachment of the inner membrane from the cell wall and by a formation of a large inter-membrane vacuolated area. In certain cells, a large of iron-binding polysaccharide was observed, which encrusted the envelope (Fig. 4d). In detail (Fig. 4e), the secretion of EPS occurred from the outer membrane to the outside in bundles, instead of in a diffusive way. A detail of the EPS emission showed that the latter is originated from nano-vesicles secreted from the outer membrane. This late overproduction of EPS is in agreement with the analytical data of total carbohydrate levels during BAS-10 growth.

A simple side experiment was performed to demonstrate that this total iron-encrusting phenomenon could be a long-term survived strategy in habitats with high iron concentrations. The BAS-10 strain was retrieved after 1 year of starvation in a spent medium by inoculating aged cells (1/100; v/v) in to a well-aerated nutrient broth: an intense growth (5·23 ± 0·28 × 10^9^ cells per ml) was observed after overnight incubation at 28°C. The growth was fast and abundant in aerobic mode, there was no growth in fresh FeC medium under anaerobic conditions. The evidence of a strong binding between Fe(III) to capsular EPS seems to have a physiological importance for cell survival under high iron concentration in anaerobic environments. So Fe(III)-EPS was further investigated by physical chemical analysis.

### Fe(III) binding to EPS

The determination of total EPS was performed in BAS-10 strain growing in FeC and NaC media. Dried and purified EPS from the NaC medium cultures was weighed at 0·25 g l^−1^ (dw) and 6·65 g l^−1^ (dw) from the FeC medium after hydrogel formation. The content of total iron in Fe(III)-EPS was determined at 35·76 ± 4·55% (dw). This percentage was higher than in commercial Fe(III)-citrate (Fluka) with Fe = 18–20%. So a calculation of total iron in 50 mmol l^−1^ Fe(III)-citrate in FeC medium was 2·71 g and we recovered 2·27 g in Fe(III)-EPS. Therefore, 84% of added iron was in the EPS, and the rest was in the FeC solution in the reduced form.

The organic component of this hydrogel is a polysaccharide and its structure was recently determined (Leone *et al.* 2007). The base molecule is a heptasaccharide repeating unit built of four rhamnose, two glucuronic acid and one galactose subunits. Molecular weight calculation of a single heptamer resulted as 1225·12 by summing each single carbohydrate. Stoichiometric calculations provided a ratio of 1 : 12, of heptamer : Fe, which corresponded to an average ratio of 1·7 iron for each sugar moiety, so the metal-to-ligand ratio was about 1 : 0·5.

**Figure 5:**
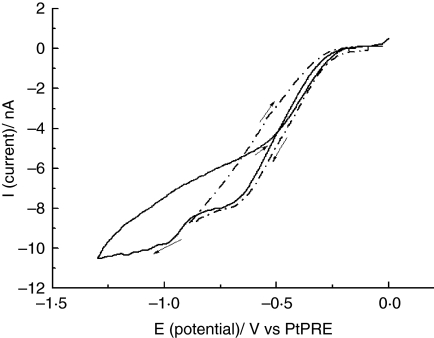
Cyclic voltammograms performed with a Hg-coated Pt microdisk 12·5 *μ*m radius in the iron hydrogel; (broken line) end potential −0·8 and −1·3 V (solid lane). Scan rate 10 mV s^−1^.

In order to shed light on the binding capacity of Fe(III) to polysaccharide and to estimate its relevant stability constant, a series of voltammetric experiments was performed directly on the ferric hydrogel. Voltammograms were recorded with a Hg-coated Pt microelectrode immersed directly in the Fe(III)-hydro gel matrix (Fig. 5) They displayed a main sigmoidal wave located at about −0·5 V (*vs* PtPRE) and a smaller one at about −0·75 V, as expected for microelectrodes working under steady-state (Montenegro *et al.* 1991; Fig. 5 solid line) No nucleation loop or stripping peak was observed in the reverse scan when the potential scan was ended after the first plateau (Fig. 5, broken line) this feature suggests that over the latter potential zone, a soluble Fe(II) species was formed during the cathodic scan (Bard and Faulkner 2001). A nucleation loop was instead observed when the forward potential scan was extended to higher negative potentials (Fig. 5 solid line), suggesting the formation of metallic iron or an insoluble species adsorbed onto the electrode surface.

**Figure 6:**
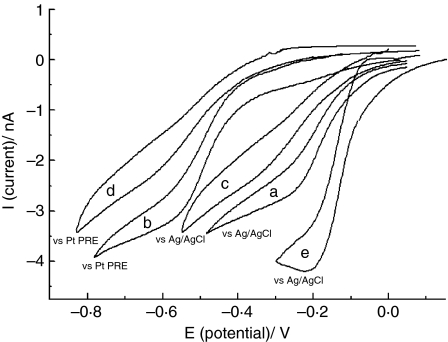
Cyclic voltammograms of 1 mmol l^−1^ Fe(III)-citrate in aqueous solutions containing 0·1 mol l^−1^ NaNO_3_ (curves a and b) and 5 mg per 20 ml polysaccharide (curves c and d), 2 mmol l^−1^ EDTA (curve e). Working electrode: Hg-coated Pt microdisk 12·5 *μ*m radius; reference electrode as indicated in the figure. Scan rate 10 mV s^−1^.

The formation of a Fe(II) soluble species at less-negative potential values is congruent with the formation of soluble Fe(II) ions in a shaken BAS-10 culture. In order to verify the nature of the chemical species responsible for the reduction wave recorded in the hydrogel, a series of voltammetric measurements were also carried out on synthetic aqueous solutions containing 1 mmol l^−1^ Fe(III)-citrate salt and NaNO_3_ as a supporting electrolyte to which different amounts of purified EPS without iron were added. In all cases, the solution pH was over the range of 5–6. Cyclic voltammograms recorded in Fe(III)-citrate solution alone, either against the Ag/AgCl electrode (Fig. 6a) or PtPRE electrode (Fig. 6b) displayed a drawn-out wave characterized by a half-wave potential (Bard and Faulkner 2001) of −0·16 V (*vs* Ag/AgCl) or −0·48 V (*vs* PtPRE). The position and general shape of this is congruent with that reported in the literature for the reduction process of Fe(III)-citrate to Fe(II)-citrate species (Battistini and Lopez-Palacios 1994; Engelmann *et al.* 2003). Upon addition of different amounts of purified EPS without iron (up to 6 mg) to the Fe(III)-citrate solution, the entire voltammetric wave shifted towards more negative potentials against Ag/AgCl (Fig. 6c) and against PtPRE (Fig. 6d), indicating that EPS binds Fe(III) ions. In addition, the latter wave was even more drawn out than that of Fe(III)-citrate indicating the occurrence of a less reversible heterogeneous electron transfer process (Bard and Faulkner 2001). Interestingly, the voltammetric wave, recorded in Fe(III)-citrate/EPS solution against PtPRE (Fig. 6d) was very close to that recorded directly in ferric hydrogel (Fig. 5, broken line). Therefore, it can conceivably be hypothesized that in both hydrogel and aqueous solution, the redox process under examination involved the same iron species, that is the reduction of Fe(III)-EPS to an undefined Fe(II) species or an Fe(II)-citrate complex.

In order to asses whether stronger complexing agents would provide sensitive changes in the voltammograms, EDTA (2 mmol l^−1^) was used as a ligand and was added to the solutions containing either Fe(III)-citrate alone or mixtures of Fe(III)-citrate and polysaccharide. Under these conditions, a well-defined process located at −0·120 V was evident (Fig. 6e). This process was similar to that attributed to the reversible reduction of Fe(III)-EDTA to Fe(II)-EDTA (Shimizu *et al.* 2007).

## Discussion

Citrate is co-transported with two Na^+^ in *Kl. pneumoniae* (Lolkema *et al.* 1994; Pos and Dimroth 1996). This feature has probably diffused to different strains belonging to closer genus such as *Kl. oxytoca* BAS-10, which grow under anaerobic conditions with citrate as a sole carbon and energy source. In *Kl. pneumoniae*, Na^+^, from sodium-citrate, plays an important role in pumping out H^+^ under anaerobic conditions and producing ATP, because it needs energy to transform oxalacetate to pyruvate by the enzyme oxalacetate decarboxylate (Bott and Dimroth 1994; Bott 1997). This energizing antiporter mechanism suggests that, in *Kl. oxytoca* BAS-10, also growing on Fe(III)-citrate, the ferric reduction to Fe(II) during citrate fermentation might replace the Na^+^ antiporter as the result of a selective adaptation for living in an iron-rich environment such as mine acid drainages as other tested strains of *Kl. oxytoca* did not thrive on Fe(III)-citrate. The presence of high concentrations of Na^+^ in FeC medium, because of buffering procedures, demonstrated that Na^+^ is favoured in respect to Fe(III) in citrate fermentation, as iron reduction is lower (10%) in respect to the growth in FeKC medium, where Na^+^ was replaced by K^+^. In this case, Fe(III) reduction was more evident (44·7%). This reduction is probably because of a ferric-reductase with a low affinity, which has been determined in whole cells and in membrane pellets (data not shown). So, Fe(III) reduction are somehow involved in citrate fermentation by BAS-10, but this feature should be investigated in more detail. However, Fe(III) reduction has been commonly observed in co-activity of sugar and aminoacid fermentation by *Escherichia coli*, *Clostridium pasteurianum* and *Lactobacillus lactis*, and in other species (Ehrlich 1996). Benz *et al.* (1998) found that *Propionibacterium freudenreichii* and other bacteria ferment humic acids and lactate by reducing amorphous Fe(III). A strain of *Clostridium bejerinckii* was instead found to ferment better glucose to butyric acid and H_2_ in the presence of higher concentrations of Fe(III)-citrate more effectively (Dobbin *et al.* 1999). Nevertheless, in this study, we report the highest percentage of ferric reduction, especially in FeKC medium, for a fermentative process.

The BAS-10 under fermenting mode by using citrate as a sole energy and carbon source, in this study had a good correlation between cell number and proteins synthesis, nevertheless proteins tended to decrease with time in favour of polysaccharide production. In the late growth phase, there was a secondary production of EPS that was concomitant with total iron precipitation in the form of a hydrogel. Fe(III)-citrate stimulates EPS production by BAS-10 cells in FeC by 26 times more than in NaC medium. The physiological reason of hyperproduction of Fe(III)-EPS could be explained by an iron storing mechanism, for example, it has been assumed that, *Shewanella oneidensis*, a Fe(III)-respiring bacteria, produces a Fe(III)-solubilizing organic ligand to supply ferric ions to the cell (Taillefert *et al.* 2007).

Direct TEM observations of BAS-10 cells suggested another possibility that during the bacterial growth, an important morphological transformation of cells occurred and caused the hydrogel formation. In the last phase, in FeC medium only, cell morphology changed from long rods to short rod with heavily iron-encrusted walls, followed by the detachment of the inner membrane and by a strong electron dense EPS extrusion from the outer membrane, indicating an important change in cell physiology of older culture. This viable but ‘nonculturable’ stage is a survival strategy reported for adverse environmental conditions, where strains decrease the size and show lower metabolic rates (Roszak and Colwell 1987; Colwell 2000). EPS later production became a strategy to undergo *vs* a dormant stage. The increase of EPS *vs* proteins in our older culture it is characteristic found in sludge settlings (Shin *et al.* 2000, 2001). EPS are believed to play a role in the binding and formation of microbial flocs. So, BAS-10 in old spent medium reduced in size; it does not undergo cell division and become nonculturable, even if are inoculated in fresh FeC medium. Conversely, cells became culturable, when a drastic environmental change occurs; bacteria are inoculated from spent FeC medium to nutrient broth, from anaerobic to aerobic conditions from high to low iron concentrations.

This new cell morphology might be considered an adaptation strategy adopted by the BAS-10 strain to protect itself from high iron concentrations in an acidic environment such as pyrite mine drainage, but this also functions in the FeC medium with acetic acid produced during citrate fermentation (Baldi *et al.* 2001). Cells retrieval in nutrient broth after a long starving period might confirm this hypothesis. However, cell iron encrustation is commonly observed mainly in iron and manganese oxidizing bacteria (Banfield *et al.* 2000; Schieber and Glamoclija 2007). In this study, iron-encrusted cells are instead found under anaerobic conditions, during a fermentative activity.

The determination of the stability of the Fe(III)-EPS complex hence become important from a physiological and biotechnological points of view. The metal-to-ligand stoichiometry was estimated to be 1–0·5. Moreover, from the voltammetric behaviour, it can be concluded that the number of binding sites in the polysaccharide was large enough to allow a complete complexation of the Fe(III) species, which was subtracted from the citrate complex. With this information, the Fe(III)-EPS-binding strength was estimated from digital simulation of the electrode process, using the commercial package DigiSim®. In this programme, equilibrium constants of homogeneous chemical reactions associated to the heterogeneous electron transfer process can be changed to obtain shape and positions of the voltammograms close to the experimental ones. By applying this procedure and on the basis of the half-wave potential change observed in the wave position (Bard and Faulkner 2001) upon addition of purified EPS to the Fe-citrate system in the aqueous solutions (i.e. from −0·06 to 0·120 V), it was found that the stability formation constant of Fe(III) with one repeating unit (heptamer) of EPS was of about one or two order of magnitude larger than that of the Fe(III)-citrate complex. For the latter species, the stability constant is *K* = 10^11^ (Battistini and Lopez-Palacios 1994; Konigsberger *et al.* 2000) and consequently *K* = 10^12^–10^13^ can be assumed to apply for the Fe(III)-EPS complex. This result corroborates the above claim concerning the stability of the Fe(III)-EPS, which may be of the same order of magnitude of Fe(III)-citrate system, but much less than that of the Fe-EDTA complex (*K* = 10^24^; Shimizu *et al.* 2007).

The strong Fe(III) binding of EPS and its sugar composition suggests several applications in all fields were iron-ligands are used such as supplying crops with cheap ferric combined molecules were iron is growth limiting, in Fenton’s type reactions to degrade aromatic compounds, as a heterogeneous catalyst, in removing NOx and SOx to clean gases, up to prevention of arthritis and iron supply for anaemic persons, and simply this EPS is itself valuable, because contains rhamnose, which represent 53·6% of all sugars.
